# Overview of Non-Cirrhotic Portal Hypertension in Pediatric Patients

**DOI:** 10.3390/jcm15176901

**Published:** 2026-09-06

**Authors:** Ambika Walecha, Senthilkumar Sankararaman, Kadakkal Radhakrishnan

**Affiliations:** 1 Lady Hardinge Medical College, New Delhi 110001, India; researchambika@gmail.com; 2 Cleveland Clinic Children’s Hospital, Cleveland, OH 44122, USA; radhakk@ccf.org

**Keywords:** non-cirrhotic portal hypertension, porto-sinusoidal vascular disease, nodular regenerative hyperplasia, obliterative portal venopathy, genetic liver disorders, immune-mediated liver injury, vascular liver disorders

## Abstract

Non-cirrhotic portal hypertension (NCPHT) is defined as portal hypertension (PHT) occurring in the absence of cirrhosis. Major etiological causes of NCPHT include immunological disorders, chronic infections, exposure to medications or toxins, prothrombotic conditions, and several genetic syndromes, highlighting that NCPHT is not a single disease but a shared phenotype arising from diverse underlying pathways. NCPHT is frequently misdiagnosed, largely due to inconsistent nomenclature and limited scientific literature. The broader term non-cirrhotic portal fibrosis (NCPF) or porto-sinusoidal vascular disease (PSVD) includes patients in a preclinical stage who demonstrate histological features similar to NCPHT but lack clinical evidence of PHT. Early detection is linked to a favorable prognosis and improved clinical outcomes. Management strategies in pediatrics continue to rely on extrapolations from adult practice, with sparse evidence to guide pediatric care. Liver biopsy remains the cornerstone of diagnosis, demonstrating nodular regenerative hyperplasia, obliterative portal venopathy, or incomplete septal fibrosis. Management focuses on prophylactic and symptomatic care, with endoscopic therapy for controlling variceal bleeding. Porto-systemic shunts and, ultimately, liver transplantation therapies may be needed for advanced stages. A pressing need exists for standardized diagnostic criteria and multicenter studies to define natural history, refine risk stratification, and evaluate therapeutic approaches in the pediatric population. This review provides an overview of the pediatric causes of NCPHT, outlines the current understanding of pathophysiology, and discusses the clinical presentations and management strategies, while highlighting existing research gaps.

## 1. Introduction

**Figure 1:**
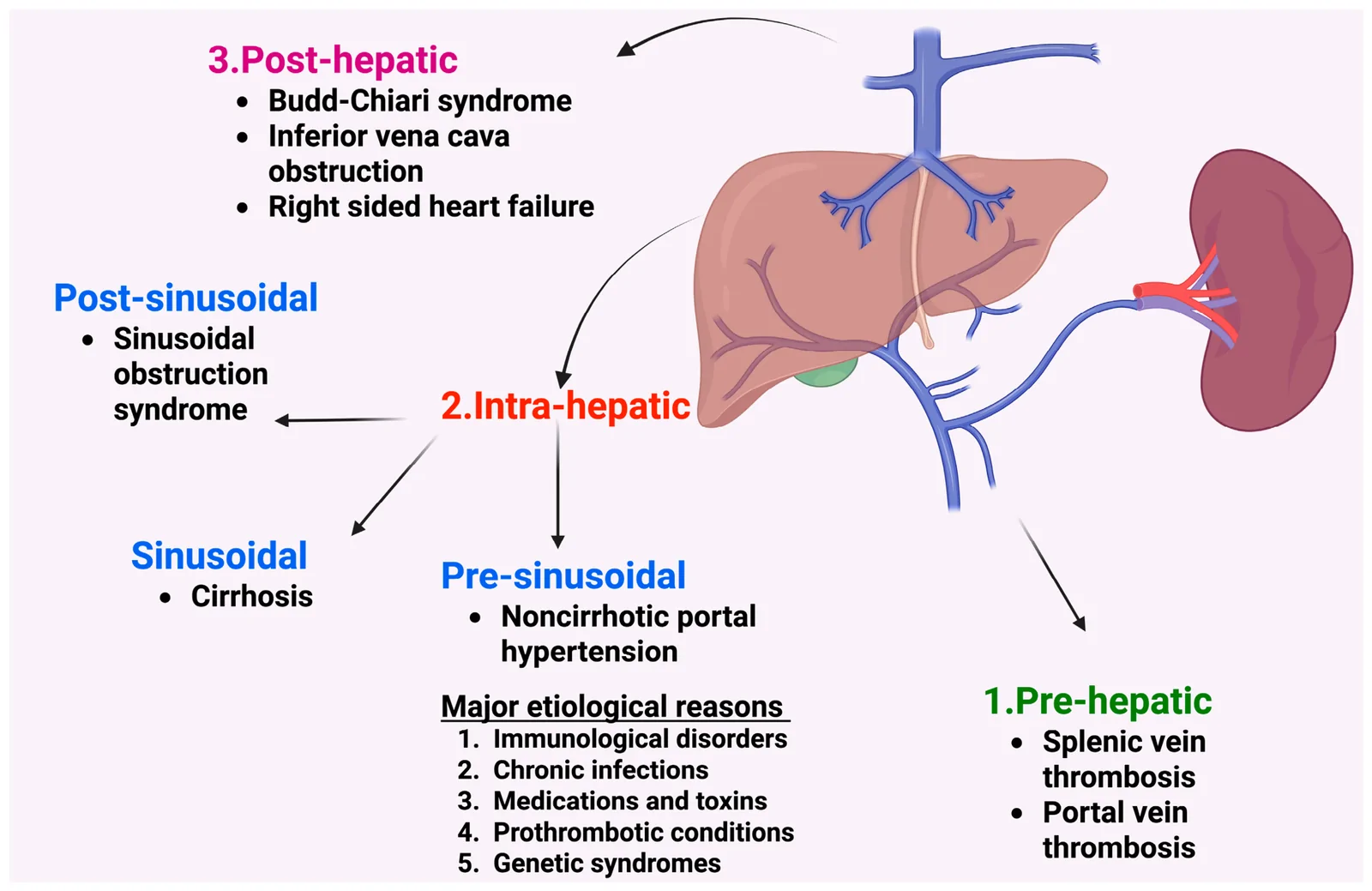
Anatomical classification of portal hypertension based on the level of vascular obstruction. Portal hypertension is categorized into prehepatic, intrahepatic, and posthepatic causes. The major etiological reasons for non-cirrhotic portal hypertension are also listed. Created in BioRender. Sankararaman, S. (2026) https://BioRender.com/mxufm74, accessed on 25 August 2026.

Portal venous pressure depends on splanchnic blood inflow and the resistance opposing this flow [1]. It is expressed as the pressure gradient between the portal vein (reflecting blood inflow into the liver) and the inferior vena cava (demonstrating venous outflow from the liver) [1]. In healthy participants, this hepatic venous pressure gradient (HVPG) ranges between 1 and 5 mm Hg. Portal hypertension (PHT) is defined as a gradient above 5 mm Hg [1,2,3,4,5,6,7]. The complications of PHT, such as variceal bleeding, usually develop when this gradient is above 10 mm Hg [8,9,10]. PHT can generally be classified as prehepatic, hepatic, and posthepatic based on the anatomical level of obstruction (Figure 1) [1,11,12,13].

Non-cirrhotic portal hypertension (NCPHT) includes a heterogeneous group of chronic vascular diseases that are characterized by elevated pressure in the portal circulation in the absence of cirrhosis [11,14,15]. Causes of NCPHT can be categorized as prehepatic (e.g., splenic or portal vein thrombosis, also referred to as extrahepatic portal vein obstruction), intrahepatic, and posthepatic (e.g., Budd–Chiari syndrome, inferior vena cava obstruction, and cardiac dysfunction) [11,13,16]. Intrahepatic causes of NCPHT can be further divided into presinusoidal and postsinusoidal (e.g., sinusoidal obstruction syndrome) [15]. The major etiological reasons for intrahepatic, presinusoidal NCPHT include immunological disorders, chronic infections, exposure to drugs (medications) or toxins, prothrombotic hematological conditions, and several genetic syndromes (Figure 1) [15,17].

With advances in molecular and genetic testing methods, an increasing number of cases previously labeled as “idiopathic NCPHT” are now attributable to specific underlying disorders, resulting in a progressively smaller proportion of truly idiopathic cases compared to historical literature in this field. Further, these disorders are frequently overlooked due to their rarity, non-specific clinical presentation, heterogeneity of the underlying conditions, and the lack of a unified approach in nomenclature and sparse management guidelines in the pediatric population [18,19,20]. As other causes of PHT, such as splenic/portal vein thrombosis and cirrhosis of the liver, are relatively more common and well described in the pediatric population, we aim to focus only on intrahepatic, presinusoidal disorders of NCPHT and hereafter will simply refer to them as NCPHT [21,22,23,24].

In this narrative review, we outline the current understanding of the pathophysiology and etiology of NCPHT, highlighting the key genetic associations, discussing the clinical presentation and management strategies, and reviewing existing research gaps. In this review, we intend to provide a broader overview of this relatively rare, heterogeneous group of diseases predisposing to NCPHT. Key differences between NCPHT and cirrhosis are also detailed in this review.

Clinicians should be aware of various terminologies that overlap with NCPHT [25]. In most Asian countries, the terms non-cirrhotic portal fibrosis (NCPF) and idiopathic portal hypertension (IPH) have been in use to describe intrahepatic disorders characterized by PHT in the absence of cirrhosis and without an identifiable cause [26]. The term “NCPHT” necessitates the inclusion of only patients with established PHT in the advanced stages. However, in the early disease process, identical microscopic changes can be demonstrated without clinically evident PHT, and this terminology does not account for these patients. Some of the prominent histological changes of NCPHT were historically referred to as nodular regenerative hyperplasia (NRH) and obliterative portal venopathy (OPV). NRH is considered an outcome of intrahepatic vasculopathy, arising from ongoing hepatocyte injury and regeneration, leading to nodule formation that compresses adjacent sinusoids and small veins, or to perisinusoidal fibrosis, changes that may progress to PHT [27]. In OPV, the pathological hallmark is injury to the portal venules and sinusoids in the absence of cirrhosis [12,28]. Other overlapping terminologies that describe the histological features include “hepato-portal sclerosis”, “non-cirrhotic portal fibrosis” and “incomplete septal fibrosis” [29]. An umbrella term, “porto-sinusoidal vascular disease” (PSVD), was suggested by De Gottardi and colleagues, highlighting that all lesions involved portal venules or sinusoids without the clinical requirement of PHT [25,30,31]. Slight differences in all these terminologies across major clinical practice guidelines do exist, and clinicians should be aware of these changes [31,32,33].

We searched PubMed using the following keywords: “non-cirrhotic portal hypertension”, “non-cirrhotic portal fibrosis”, “idiopathic portal hypertension”, “porto-sinusoidal vascular disease”, “nodular regenerative hyperplasia”, and “obliterative portal venopathy”. We prioritized the inclusion of pediatric studies, supplemented by adult data where pediatric literature remains sparse.

## 2. Epidemiology

Given the rarity of NCPHT and the widespread heterogeneity in its nomenclature, accurately determining its epidemiology in all age groups remains difficult. It is estimated that NCPHT accounts for approximately 15–34% of all cases of PHT, with a noticeably higher prevalence in Japan and the Indian subcontinent, where it constitutes nearly one-third of all cases across all age groups [34,35]. The overall prevalence is declining, likely due to improvements in sanitation and hygiene [36]. In Western countries, it generally represents up to 5–16% of all PHT cases [16,20,37].

Epidemiological patterns also show geographic variation in gender distribution across age groups. Most adult cohorts from the West and the Indian subcontinent report a male predominance, whereas Japanese cohorts demonstrate a higher prevalence among females. The mean age of patients in the Indian cohort is 25–35 years, whereas Japanese and Western cohorts report a higher mean age of 36–56 years [20,37]. Single-center pediatric studies noted an average age between 5 and 12 years [38,39]. To our knowledge, no large-scale, population-based studies have been published specifically assessing NCPHT in the pediatric population.

## 3. Pathophysiology

A dual theory has been postulated to unravel the largely unknown and poorly characterized pathogenesis of this complex disease [12]. The first theory, popularly referred to as the “intrahepatic vascular obstruction” (resistance-initiated) theory, states that NCPHT can be triggered by any factor that injures the intrahepatic vascular bed. These include hypercoagulable states such as thrombophilia or antiphospholipid antibodies; T-cell-mediated autoimmune endothelial damage; and infections that may lead to microthrombosis [19,27,40,41]. This injury to the vascular bed may promote the occlusion of small portal veins, leading to liver ischemia. As a result, some areas of the liver undergo atrophy, whereas other regions develop compensatory hypertrophy. This structural distortion raises resistance to portal blood flow, ultimately leading to elevated portal pressures [40].

The second theory postulates overexpression of iNOS and eNOS in patients with NCPHT. These enzymes are responsible for nitric oxide (NO) synthesis; their overactivity results in sustained local NO overproduction. NO, being a potent vasodilator, causes dilation of the splenic sinusoids, resulting in progressive splenomegaly and a marked increase in splenic blood pooling. The enlarged and hyperperfused spleen augments portal inflow, thereby contributing directly to elevated portal pressures. As per this hypothesis, in NCPHT, splenomegaly may not simply be a passive consequence of vascular congestion but could be a pathogenic driver of the disease process. However, due to the limited and inconclusive nature of current evidence, this hypothesis remains speculative and requires further validation through robust studies [41].

## 4. Underlying Etiological Conditions Implicated in NCPHT

The underlying etiology of NCPHT is broadly categorized into five major groups: (i) immunological disorders, (ii) prothrombotic conditions, (iii) chronic infections, (iv) genetic syndromes, and (v) exposure to medications or toxins [17,41]. Although these associations are mostly better characterized in adult cohorts, it is reasonable to assume that pediatric NCPHT arises through a similar multifactorial interplay.

### 4.1. Immune-Mediated and Autoimmune Disorders

Immunological changes, such as decreased CD8^+^ cells and an altered CD4/CD8 ratio, are well described in OPV and play a crucial role in the pathogenesis of NCPHT [37]. Corroborating evidence for immune-mediated pathogenesis also comes from liver involvement in pediatric common variable immunodeficiency (CVID). CVID is associated with immune dysfunction that often manifests as NRH [42,43]. Persistent cytotoxic CD8^+^ T-cell infiltration targeting sinusoidal endothelial cells, along with alterations in the intestinal microbiome leading to translocation of inflammatory bacteria and endotoxins, constitutes the immunological basis for the development of liver pathological features such as PSVD and NRH in CVID patients [42]. These combined effects of endothelial-directed cytotoxicity and a disrupted gut–liver axis can ultimately lead to significant liver dysfunction. Similar mechanisms have been described in people (both pediatric and adult patients) with inborn errors of immunity [44], including ataxia telangiectasia [45], adenosine deaminase 2 (ADA2) deficiency [46], and chronic granulomatous disease [47]. Immunodeficiency-associated hepatic histology and clinical outcomes are noted in Table 1.

### 4.2. Prothrombotic Disorders

One of the major causes of PHT in the pediatric age group is extrahepatic portal vein obstruction (EHPVO), which is most often due to portal vein thrombosis triggered by factors such as umbilical sepsis, catheter-related infections, or neonatal interventions [22]. However, this represents extrahepatic thrombosis and is distinct from NCPHT, which is an intrahepatic disorder [17]. Nevertheless, both conditions can share an underlying prothrombotic state, resulting in a spectrum of manifestations [12]. The unifying hypothesis proposed by Sarin and Kumar suggests that a major thrombotic event early in life tends to occlude the main portal vein, leading to EHPVO, whereas repeated microthrombotic events later in life result in the gradual obliteration of small and medium intrahepatic portal venous branches, leading to NCPHT [49]. Supporting this concept, findings from a Western cohort showed that many adult patients with NCPHT have occult prothrombotic disorders, including myeloproliferative diseases, protein C/S deficiency, and antiphospholipid antibodies. These conditions promote tiny, recurrent microthrombi in intrahepatic portal venules, progressively narrowing or blocking them and producing OPV, one of the hallmarks of NCPHT [50].

### 4.3. Chronic Infections

Abdominal infections, particularly in early life, have long been implicated in the pathogenesis of OPV and the broader PSVD/NCPHT spectrum, especially in socioeconomically disadvantaged settings where recurrent bacterial exposure is common [19]. In infants, abdominal infections at birth, including omphalitis, neonatal sepsis, and recurrent diarrheal illnesses, are thought to trigger portal pyemia and pylephlebitis, setting off cycles of endothelial injury, microthrombi, and sclerosis of small- and medium-sized portal vein branches. This ultimately evolves into the characteristic vascular remodeling of OPV [37]. Experimental animal studies have supported this infectious hypothesis, as injecting dead *E. coli* or non-pathogenic colon bacilli into the portal circulation of animals reproduced IPH-like changes [40].

### 4.4. Genetic Syndromes

Many syndromes have been implicated in NCPHT, and the common ones are described here. 

#### 4.4.1. Turner Syndrome

Recent studies identified liver function abnormalities and three principal patterns of liver involvement, namely steatosis, fibrosis, biliary lesions, and vascular-mediated architectural changes such as NRH and focal nodular hyperplasia, in Turner syndrome in both pediatric and adult patients [51,52]. The authors suggested that Turner syndrome is characterized by congenital vascular malformations and OPV, which alter the intrahepatic blood flow and promote the development of NRH. Another study in adults with Turner syndrome revealed that approximately one-fourth of the cases showed NCPHT-like histology, with three patients progressing to clinically significant PHT [53].

#### 4.4.2. Adams–Oliver Syndrome

Adams–Oliver syndrome is a congenital vascular disorder; patients typically present with aplasia cutis, cutis marmorata, and limb anomalies such as syndactyly and absent phalanges. Several pediatric cases have reported the presence of NCPHT among Adams–Oliver syndrome patients, and experts highlight that Adams–Oliver syndrome and NCPHT share a common mechanism involving congenital microvascular abnormalities and a predisposition to thrombosis, which may be modulated genetically [54,55].

#### 4.4.3. Prolidase Deficiency

Prolidase deficiency leads to impaired wound healing due to its important role in collagen turnover. Reduced activity of the enzyme leads to characteristic clinical features such as facial dysmorphism, recurrent infections, and splenomegaly, and a potential link to vascular-mediated liver injury [56]. In contrast, excess prolidase activity has been linked to several fibrotic liver diseases, whereas its deficiency could potentially contribute to vascular-mediated liver injury. These effects may reflect impaired angiogenesis and aberrant inflammatory signaling, such as nuclear factor kappa B (NF-κB) activation. Consistent with these findings, a recent case series in adults also suggested that prolidase deficiency may predispose to portal venous abnormalities and NCPHT [57].

#### 4.4.4. Short Telomere Disorders

Dyskeratosis congenita and the broader telomere biology disorders represent a group of conditions marked by markedly shortened telomeres. These are clinical syndromes arising from telomere dysfunction, with hallmark physical features including nail dystrophy, oral leukoplakia, reticular skin, and bone marrow failure. Younger individuals typically present with bone marrow failure, immunodeficiency, mucocutaneous stigmata, and gastrointestinal inflammation, whereas adults typically have isolated marrow failure, pulmonary fibrosis, or liver disease. In a recent cohort of 58 patients (both pediatric and adult age groups) with dyskeratosis congenita/telomere biology disorder, around 10 patients (17.2%) developed clinically significant PHT over a seven-year follow-up [58]. Detailed histology revealed that three patients had a definite non-cirrhotic PHT pattern, characterized by nodular regenerative hyperplasia with OPV and periportal or bridging fibrosis. In addition, a subset of patients showed mild periportal/pericellular fibrosis or hemosiderosis with clinically established PHT. These constellations of findings are compatible with early or presumed PSVD/NCPHT. Overall, the study highlighted that patients with dyskeratosis congenita/telomere biology disorders, particularly those with severe multisystem phenotypes and autosomal recessive/X-linked recessive or TERF1-interacting nuclear factor 2 variants, may develop NCPHT, with a spectrum of histological changes [58].

#### 4.4.5. Cystic Fibrosis

Liver involvement in the form of advanced cystic fibrosis (CF)-related disease (aCFLD), characterized by cirrhosis of the liver, has been reported in approximately 5–10% of people with CF, with onset of presentation in the first decade of life [59,60]. On the other hand, in the later adult age group, NCPHT has been documented in several case studies [59,61,62,63]. Platelet activation, a pro-inflammatory state characterized by vasculitis (endothelialitis), and recurrent infections (pulmonary and intestinal) have been postulated to be risk factors for NCPHT along with CF transmembrane conductance regulator (CFTR) dysfunction in CF [59].

#### 4.4.6. The Genetic and Epigenetic Landscape

Numerous genetic and epigenetic studies have identified molecular defects that may lead to progressive injury and remodeling of the portal microvasculature, which are detailed in Table 2. These pathways outline a genetic landscape in which vascular, metabolic, and immune factors interact to give rise to an NCPHT phenotype. On a case-by-case basis, genetic screening for these abnormalities, especially in early-onset, syndromic, or familial cases, may support a timely underlying diagnosis and informed, individualized care. Future research should validate these associations and evaluate potential therapeutic targets for this disorder.

### 4.5. Drugs and Toxins

Medications such as 6-thioguanine have been implicated in causing NCPHT through their ability to cause microvascular injury and induce NRH. For example, Rawat et al. followed 10 children with 6-thioguanine toxicity and found that most had splenomegaly, thrombocytopenia, and PHT long after stopping the drug [72]. Moulik and Taj similarly reported that 8 of 11 children on 6-thioguanine developed features of PHT over 10 years [73]. These studies underscore the importance of long-term surveillance in patients treated with thiopurines. Other implicated drugs/toxins in NCPHT pathogenesis from adult cohorts include arsenic compounds [74], other chemotherapy medications such as oxaliplatin [75], and antiretroviral therapies [76], though the pediatric data remain sparse.

## 5. Clinical Approach to NCPHT

### 5.1. Clinical Manifestations

In pediatric patients, NCPHT commonly presents in the first two decades, whereas in adults it is typically present in the third or fourth decade of life [16,49,77]. Asymptomatic splenomegaly and complications of splenomegaly, such as hypersplenism, are more commonly reported in children than in adults [27,78,79]. Liver function is generally preserved, and in later stages, patients may show derangements in liver enzymes, prothrombin time, and albumin [43]. Episodes of variceal bleeding also occur, and this complication may increase with age [16,79]. Nearly half of patients develop ascites following variceal bleeding or infection, which is easier to manage compared to cirrhosis [80]. A small subset of patients may also develop portal vein thrombosis and, rarely, hepatic encephalopathy [19]. Features such as reduced physical activity, abdominal discomfort from splenomegaly, and psychosocial concerns are well described in children with EHPVO, and similar issues may also be observed in pediatric NCPHT, although dedicated studies in this population are limited [78,81].

### 5.2. Diagnostic Approach to NCPHT

NCPHT should be suspected in patients who present with signs of PHT but maintain preserved liver synthetic function and lack a defined cause of chronic liver disease. A thorough and systematic diagnostic workup integrating clinical assessment, laboratory studies, imaging, and histology is essential to exclude alternative etiologies and to support an accurate diagnosis of NCPHT. Owing to the diagnostic challenges, many criteria have been proposed for adults with NCPHT, including the Japanese criteria, the Asian Pacific Association for the Study of Liver recommendations, and the framework by Schouten et al., which are listed below [41].

The Japanese criteria emphasized splenomegaly, anemia, and signs of PHT, with no evidence of cirrhosis, hematologic disease, parasitic infection, or vascular obstruction [82].

The Asian Pacific Association for the Study of Liver criteria required preserved liver function, normal hepatic and portal vein patency, and histologic exclusion of parenchymal liver disease, while ruling out causes such as viral hepatitis, metabolic-associated liver disease, and schistosomiasis [31].

Schouten et al. proposed a comprehensive set of criteria that incorporated five components for diagnosis, including clinical PHT, histological absence of cirrhosis, exclusion of chronic parenchymal liver disease, exclusion of other causes of NCPHT, and normal vascular patency [19].

A fundamental diagnostic challenge is to differentiate NCPHT from cirrhosis, given their similarity in clinical presentation and potential for misdiagnosis [83]. The spectrum of phenotypic concordance between NCPHT and cirrhosis includes splenomegaly and variceal bleeding. Sometimes, patients are mislabeled as having cryptogenic cirrhosis. Routine imaging modalities cannot reliably differentiate NCPHT from early cirrhosis. Since no imaging finding is pathognomonic, liver biopsy remains a key diagnostic modality [83]. The salient differences between NCPHT and PHT secondary to cirrhosis are noted in Table 3.

### 5.3. Laboratory Investigations

Complete blood count may show pancytopenia due to hypersplenism, and anemia can be worsened by recurrent variceal bleeding [12,37]. Serum albumin, liver enzymes, and prothrombin time are usually normal in early stages [12,37]. The laboratory investigations that are commonly performed to evaluate the underlying causes for NCPHT and cirrhosis with PHT are detailed in Table 4.

### 5.4. Radiological Tests

Abdominal ultrasound with Doppler interrogation of the portal vasculature is the first-line radiological test in NCPHT. Imaging typically shows a normal-sized liver and echotexture, and splenomegaly. The characteristic portal venous abnormalities may include portal vein thickening (>3 mm) with echogenic walls and smooth intrahepatic radicles [12,84]. Patients with NRH show nodularity of the liver, which is often confused with cirrhotic nodules. Contrast-enhanced cross-sectional imaging (computer tomography and magnetic resonance imaging) is further helpful to delineate intrahepatic portal vein abnormalities (non-visualization, reduced caliber, occlusive thrombosis), focal nodular liver changes, and possible perfusion defects, which may sometimes help to differentiate NCPHT from cirrhosis.

Elastography methods (transient elastography, shear wave elastography, and magnetic resonance elastography) can be used to measure liver stiffness. Even though liver stiffness measurements tend to be lower in NCPHT when compared to cirrhosis, patients with NRH may show elevated readings (sometimes up to ~22 kPa), which overlap with measurements from early cirrhosis. As liver stiffness measurement correlates poorly with fibrosis in NCPHT, it may create false reassurance or lead to misclassification. Conversely, in a pediatric study, splenic stiffness measurements were noted to be useful in INCPHT due to congestion in the spleen and alterations in splenic architecture, resulting in markedly elevated splenic stiffness [85].

Interventional hemodynamic studies with HVPG can be utilized [86]. HVPG measures pressure across the hepatic sinusoids and is therefore most reliable in sinusoidal causes of portal hypertension, particularly cirrhosis. As NCPHT is a presinusoidal disorder, the HVPG typically remains near normal values. However, in up to 30% of patients, particularly those with NRH, HVPG may be >10 mm Hg if the predominant cause of NRH is sinusoidal, thus making its diagnostic utility challenging. Unlike adults, no definitive link has been established between the degree of fibrosis, probability of complications, and HVPG; its utility remains limited for pediatric PHT. There are several limitations to using HVPG as a surrogate for portal hypertension in children. Because most adult portal hypertension is related to cirrhosis, HVPG is widely used to assess portal hypertension and is clinically significant. In contrast, Ebel et al. noted that INCPH may not accurately reflect the severity or predict complications of INCPH [86].

### 5.5. Endoscopy

Upper gastrointestinal and anorectal endoscopic evaluations commonly reveal prominent esophageal and anorectal varices, respectively, often discordant with preserved hepatic synthetic function [12,84]. Esophageal varices are commonly seen in 80–90% of cases of NCPHT, and compared to cirrhosis, both esophageal and anorectal varices tend to be generally larger.

### 5.6. Histopathological Examination of the Liver

Given that the definition of NCPHT requires the exclusion of cirrhosis and demonstration of characteristic vascular lesions, histological evaluation forms the anchor of diagnosis, invariably requiring liver biopsy for confirmation. However, the lesions in NCPHT can be subtle and can be easily overlooked, requiring an expert pathologist to ensure accurate recognition [20]. While gross examination may reveal an enlarged, normal, or shrunken liver, definitive diagnosis is established based on characteristic microscopic lesions. Histologically, lesions can be divided into specific and non-specific changes. The specific lesions include OPV, progressive narrowing of the portal vein lumen, NRH, alternating hyperplastic hepatocytes with atrophic plates, and incomplete septal fibrosis, with intersection of hepatic parenchyma by incomplete fibrotic bands near portal tracts and central veins [17].

As NCPHT is patchy in nature, liver biopsy can either underestimate or overestimate the disease severity. Some of the non-specific features that may support the diagnosis of NCPHT include herniated portal vein branches, hypervascular or periportal thin-walled vessels within or around portal tracts, sinusoidal dilatation, and mild perisinusoidal fibrosis. When such changes are identified in the absence of clinically evident PHT, they may represent a preclinical stage of the disease spectrum (referred to as NCPF, which is discussed in an earlier section) [31,37].

## 6. Management of NCPHT

Management of NCPHT in children is largely extrapolated from studies conducted in adults due to a lack of clinical trials in the pediatric age group [87,88]. Due to a lack of effective therapies targeting the central vascular pathology, the management of NCPHT primarily revolves around symptomatic treatment and prevention of complications. A major focus of management is the prevention of variceal bleeding [12]. Overall, the prognosis of NCPHT remains much better than that of cirrhosis with PHT. In a single-center pediatric study which included 40 patients with PHT (24 with cirrhosis and 16 with NCPHT), hepatopulmonary syndrome was noted in 16% of patients with cirrhosis, but none of the patients with NCPHT had hepatopulmonary syndrome [89]. Management by an interdisciplinary team consisting of a hepatologist/gastroenterologist, pathologist, hematologist, geneticist, radiologist, surgeon, immunologist, infectious disease specialist, clinical pharmacist, registered nurse, registered dietitian, and social worker/case manager is often needed based on the underlying condition. Compared to adults, the centers managing these complications may not be available in proximity to pediatric patient families, and management decisions should be thoughtfully made after detailed discussion with the families.

### 6.1. Medical Therapies

#### 6.1.1. Beta-Blockers

The pharmacological benefit of beta-blockers in PHT is due to decreased portal pressure through a reduction in portal and collateral blood flow [1]. Beta-blockers are routinely used in adults for primary and secondary prophylaxis of PHT, but their use in pediatric patients is still controversial [88,90,91]. The contraindications of beta-blockers include asthma, other pulmonary disorders, and cardiac block [92,93]. Also, clinicians should be aware of the fact that beta-blockers attenuate the sympathetic compensatory response during a bleeding episode, predisposing to hypotension, bradycardia, and masking of shock, and this may delay presentation to an emergency department [93]. Also, unlike in adults, there is a lack of target heart rate dosing guidelines in pediatrics. Hence, the utility of beta-blockers requires further research and robust clinical trials to determine their efficacy and to better define guidelines for managing pediatric NCPHT [94].

#### 6.1.2. Anticoagulation

Routine use of anticoagulants is not recommended for NCPHT. Instead, it is strictly reserved for cases secondarily complicated by portal vein thrombosis [95]. According to the Baveno consensus guidelines for adults with non-cirrhotic portal vein thrombosis, low molecular weight heparin is preferred as a first-line therapy if a patient with NCPHT develops an acute, sudden-onset clot [5]. Conversely, rivaroxaban is considered safe and effective for long-term suppression in adults if an NCPHT patient has chronic clotting tendencies or permanent thrombotic risk factors [96]. A large European retrospective cohort study in adults evaluating long-term outcomes associated with IPH documented portal vein thrombosis in approximately 40% of the patients [97]. Comorbidities, notably HIV infection, were associated with a fivefold increased risk of developing PVT. In non-HIV patients, variceal bleeding at diagnosis and higher serum bilirubin were linked to PVT. Overall, approximately half of anticoagulated patients achieved complete or partial recanalization, a rate comparable to that reported in cirrhosis [97]. These findings suggested a possible role of PHT severity and associated immune and prothrombotic mechanisms in thrombosis development [97]. Complementing these observations, a recent case report in an adult described a novel targeted approach in a patient with paroxysmal nocturnal hemoglobinuria complicated by NCPHT, using eculizumab (a C5 inhibitor), which not only controlled hemolysis but also resolved PHT symptoms [98].

### 6.2. Endoscopic Therapy

Given the reliance on symptomatic management, endoscopic therapy remains the key driving force in care for managing life-threatening variceal bleeding. A recent study evaluating endoscopic treatment options, including variceal ligation and sclerotherapy, found that both modalities have comparable success in eradicating esophageal varices and controlling bleeding [18]. However, endoscopic variceal ligation could offer notable advantages, including fewer treatment sessions, a lower risk of complications, and a reduced likelihood of re-bleeding. Notably, the study also highlighted that a sequential approach, endoscopic variceal ligation followed by endoscopic sclerotherapy, outperforms either modality alone. This combined strategy reduces the overall number of sessions, limits exposure to sclerosant, and results in fewer complications. However, children, especially those under two years, have anatomical constraints limiting the feasibility of endoscopic variceal ligation; thus, endoscopic sclerotherapy remains the more practical choice [18].

### 6.3. Interventional Radiological/Surgical Interventions

The literature on the surgical management of NCPHT in both adults and pediatric patients remains sparse. Most robust literature is derived from management of PHT from cirrhosis and extrahepatic portal vein thrombosis [1]. Also, many studies across all ages noted that patients with NCPHT may develop portal vein thrombosis as a secondary complication [14,99].

#### 6.3.1. Portosystemic Shunts and Surgical Interventions

Transjugular intrahepatic portosystemic shunt (TIPS) is an effective therapeutic option for patients with severe or refractory complications of NCPHT [100]. Compared with adult patients with cirrhosis, those with NCPHT generally may experience better outcomes following TIPS, including lower rates of hepatic encephalopathy, decompensated hepatic insufficiency, and mortality [80]. However, rates of recurrent variceal bleeding and shunt dysfunction appear to be comparable between the two groups. Careful patient selection remains crucial, with preserved renal function and the absence of significant comorbidities being important considerations before TIPS placement [95]. TIPS is technically challenging due to small vessel caliber and possible vascular anomalies in young children [101]. In a single-center study involving 26 children with a mean age of 5.2 years, nearly 50% underwent surgical interventions [99]. Seven had surgical shunts (initial shunts included a Rex shunt in four patients and a splenorenal shunt in three patients) [99]. One patient had TIPS, and four had splenectomy. The other half were managed conservatively. A recent meta-analysis underscores that TIPS placement can be highly successful in pediatric patients in centers of excellence, despite unique technical challenges [102]. These anatomical and technical limitations can be effectively circumvented by adapting modifications of general tools, using intravascular ultrasound, and implementing pediatric-specific modified techniques by appropriate specialized teams [102]. For pediatric patients with predominant spleen-related issues, a non-selective portosystemic shunt with splenectomy may be a reasonable option, though it carries risks of long-term complications of splenectomy, including increased infections [18].

#### 6.3.2. Liver Transplantation

Liver transplantation is an option in a subset of NCPHT patients with life-threatening complications such as decompensated liver failure, hepatic encephalopathy, and hepatopulmonary syndrome. Early data from adult studies showed that patients with severe NCPHT achieved good outcomes after liver transplantation [103]. Interestingly, some were misdiagnosed as having cirrhosis, but explant livers showed NRH and focal fibrosis, highlighting how frequently NCPHT is mistaken for cirrhosis [103]. A recent case report in an adult exemplified that severe NCPHT can coexist with EHPVO, with successful management of both through liver transplantation and vascular grafts [104]. In a recent multicenter study in adults, 79 patients with PVSD requiring liver transplantation for various conditions, such as refractory ascites, encephalopathy, or hepatorenal syndrome, were analyzed [105]. The study reported worse post-transplant outcomes in those with elevated baseline bilirubin, renal impairment, and advanced PHT. Overall, these findings suggest that liver transplantation can effectively reverse NCPHT in carefully selected patients.

### 6.4. Extrahepatic Management and Emerging Targeted Therapies

In certain inherited disorders associated with NCPHT, such as short telomere syndromes, the management of extrahepatic complications becomes critical. Allogeneic hematopoietic stem cell transplant is the only curative option for bone marrow failure in these syndromes, but it carries a significant risk with advanced disease and pre-existing organ dysfunction [106]. Reduced-intensity conditioning and meticulous pre-transplant evaluation are important for reducing toxicity. While a hematopoietic stem cell transplant can reverse marrow failure, outcomes in the post-transplant setting remain limited by substantial mortality from pulmonary fibrosis, infections, and worsening of hepatic involvement [106]. Interestingly, emerging research has highlighted the role of aberrant tyrosine kinase signaling in RASopathy, a group of disorders characterized by abnormal regulation of the RAS-MAPK pathway and phenotypes overlapping with Noonan syndrome and myeloproliferative disorder. Casitas B-lineage lymphoma (CBL) protein acts as a critical E3 ubiquitin ligase and functions as a negative regulator of multiple tyrosine kinases, and its loss-of-function results in augmented downstream signaling, providing a theoretical rationale for targeted kinase inhibition, including spleen tyrosine kinase [107]. Recently, a case report described NCPHT as a novel vascular manifestation in a child with CBL RASopathy, further expanding the phenotypic spectrum of this disorder. Further studies are required to elucidate the role of tyrosine kinase-directed therapies and to define their potential relevance in managing vascular and hepatic manifestations associated with RASopathy [107].

## 7. Research Gaps and Future Research Directions

Most pediatric data are derived from single-center cohorts, and prospective multicenter studies are urgently needed. Age-specific cutoffs for noninvasive liver and spleen stiffness measurements in children are lacking and needed. Management is mostly symptomatic, with PHT (pharmacological/endoscopic therapies vs. radiological/surgical interventions), and interventions targeting the pathophysiology need much research.

## 8. Conclusions

NCPHT in the pediatric population is a relatively rare condition with limited scientific literature largely due to its heterogeneous nature and inconsistent nomenclature. The broad etiological spectrum includes immunological disorders, prothrombotic conditions, chronic infections, genetic syndromes, and exposure to medications or toxins, highlighting that NCPHT is not a single disease but a shared phenotype arising from diverse pathways. Early detection is linked to a favorable prognosis and improved clinical outcomes. Management strategies for children continue to rely on extrapolations from adult practice, with sparse evidence to guide pediatric care. A pressing need exists for standardized diagnostic criteria and multicenter studies to define natural history, refine risk stratification, and evaluate therapeutic approaches in the pediatric population. Addressing these evidence gaps is essential for earlier recognition and mechanism-based treatment in children with NCPHT.

## Figures and Tables

**Table 1:** Immunodeficiency-associated hepatic histology and clinical outcomes.

Immunodeficiency	Hepatic Histology	Clinical Significance and Outcome
CVID and XLA–cohort of 123 adult patients with primary antibody deficiencies (117 CVID, 6 XLA; mean age ~50 years). Approximately one-sixth of patients with CVID had features of NCPHT [44]	Histological findings included sinusoidal changes such as endothelialization, congestion, and dilatation, and parenchymal micronodular formation	NCPHT was associated with more severe clinical and immunological phenotypes. The authors recommend active evaluation for NCPHT in patients with primary antibody deficiencies, while also considering primary antibody deficiency in patients presenting with otherwise unexplained NCPHT
AT–rare pediatric association reported in a 6-year-old child [45]	NRH	NRH represents an uncommon hepatic manifestation of AT and may occur in addition to the more recognized hepatic abnormalities, including dyslipidemia, elevated liver enzymes, and hepatic steatosis
ADA2 deficiency–two pediatric cases with hepatic involvement [48]	NRH consistent with hepatic vascular involvement	Although neurological and hematological manifestations are well recognized in ADA2 deficiency, hepatic vascular involvement should be considered in patients with unexplained elevations in liver enzymes
Chronic granulomatous disease–cohort of 194 patients (mean age 19.3 years among surviving patients) evaluating the impact of NRH on survival [47]	NRH	Development of NRH was associated with poorer prognosis and increased mortality, suggesting that NRH may represent an important marker of disease severity in CGD

ADA2—adenosine deaminase 2, AT—ataxia-telangiectasia, CGD—chronic granulomatous disease, CVID—common variable immunodeficiency, NCPHT—non-cirrhotic portal hypertension, NRH—nodular regenerative hyperplasia, XLA—X-linked agammaglobulinemia.

**Table 2:** Genes implicated in NCPHT and their associated pathogenic pathways.

Gene	Gene Function	Clinical Impact in Abnormal Gene Function
*HRG* [64]	Coagulation, fibrinolysis, immune modulation	Microvascular injury, disruption in endothelial integrity, and NCPHT
*KCNN3* (SK3 Channel) [65]	Endothelial hyperpolarization; regulation of vascular tone	Hepatic microcirculatory dysfunction leading to NCPHT
*DGUOK* [66]	Mitochondrial DNA maintenance; purine salvage pathway	Mitochondrial dysfunction due to impaired ATP binding; similar mechanism has been implicated in didanosine hepatotoxicity
*NT5C2* and *XDH* [67]	Enzyme involved in purine metabolism; influence handling of nucleoside analogs (including didanosine)	Associated with NCPHT in HIV-positive patients on didanosine
*DOCK8* [68]	Immune regulation; leukocyte migration and survival	Reported in NCPHT with hyper-IgE syndrome and invasive fungal infections
*GIMAP5* [69,70,71]	Lymphocyte survival and endothelial cell homeostasis	Direct endothelial remodeling and development of NCPHT: capillarization of liver sinusoidal endothelial cells leads to an impaired endothelial phenotype.

ATP—adenosine triphosphate; *DGUOK*—Deoxyguanosine kinase; *DOCK8*—dedicator of cytokinesis 8; *GIMAP5*—GTPase, immune-associated protein 5; *HRG*—Histidine-rich glycoprotein; HIV—human immunodeficiency virus; IgE—immunoglobulin E; *KCNN3*—Potassium calcium activated channel subfamily N member 3; SK3—small-conductance calcium activated potassium channel 3; NCPHT—non-cirrhotic portal hypertension; *NT5C2*—5′-Nucleotidase, cytosolic II; *XDH*—Xanthine dehydrogenase.

**Table 3:** Salient differences between cirrhotic and non-cirrhotic portal hypertension.

Features	Idiopathic Non-Cirrhotic Portal Hypertension	Cirrhosis with Portal Hypertension
Splenomegaly	Early feature and asymptomatic splenomegaly. Size can be massive	Often present but usually less pronounced in early stages
Ascites, jaundice, and encephalopathy	Typically absent until the late stages. May present after a major episode of variceal bleed or post-shunt surgery.	Relatively common and may occur earlier
Gastrointestinal (variceal) bleeding	Common	Common
Stigmata of chronic liver disease (palmar erythema, spider nevi, gynecomastia, telangiectasia)	Absent	Common
Overall health status	Preserved well until late stages	Often impaired earlier in the disease course
Liver function tests	Usually preserved until late stages	Often impaired
Liver pathology	Wrinkled, irregular macroscopic liver surface, septal fibrosis, nodular regenerative hyperplasia	Disrupted architecture; fibrosis with regenerative nodules and other features as detailed earlier

**Table 4:** Laboratory tests that are commonly performed to evaluate the underlying causes for NCPHT and cirrhosis with portal hypertension.

Tests	Comments
Infectious disease workup	Viral panel for hepatitis B and C viruses, and HIV testing, stool and ova testing for schistosomiasis.
Immunological labs	IgG, ANA, smooth muscle antibodies, anti-LKM to evaluate autoimmune hepatitis
Metabolic conditions predisposing to chronic liver disease	Serum iron, iron-binding capacity, and ferritin (hemochromatosis), ceruloplasmin and 24 h urinary copper (for Wilson disease), alpha-1 antitrypsin phenotype
Thrombophilia screen to evaluate EHPVO and also for NCPHT	Factor V Leiden, protein C/S, antiphospholipid antibodies
Genetic evaluation	Targeted gene panel, whole exome sequencing, and whole genome sequencing as appropriate to evaluate NCPHT vs. other chronic liver disorders as noted earlier in the review

ANA—antinuclear antibody; anti-HCV—hepatitis C virus antibody; anti-LKM—anti-liver kidney microsomal antibody; EHPVO—extrahepatic portal vein obstruction; HBsAg—hepatitis B surface antigen; HIV—human immunodeficiency virus; IgG—immunoglobulin G; INR—international normalized ratio; PT—prothrombin time.

## Data Availability

No new data were created or analyzed in this study. Data sharing is not applicable to this study.
